# A Tar aspartate receptor and Rubisco-like protein substitute biotin in the growth of rhizobial strains

**DOI:** 10.1099/mic.0.001130

**Published:** 2022-01-25

**Authors:** Carmen Vargas-Lagunas, Yolanda Mora, Alejandro Aguilar, Alma Ruth Reyes-González, Alejandra Arteaga-Ide, Michael F. Dunn, Sergio Encarnación, Lourdes Girard, Humberto Peralta, Jaime Mora

**Affiliations:** ^1^​ Programa de Genómica Funcional de Procariotes, Laboratorio de Biología de Sistemas y Biología Sintética, Centro de Ciencias Genómicas, Universidad Nacional Autónoma de México, Cuernavaca, Mexico

**Keywords:** subcultures, methyl-accepting proteins, chemotaxis, rhizobia, nitrogen fixation, vitamins, metabolism

## Abstract

Biotin is a key cofactor of metabolic carboxylases, although many rhizobial strains are biotin auxotrophs. When some of these strains were serially subcultured in minimal medium, they showed diminished growth and increased excretion of metabolites. The addition of biotin, or genetic complementation with biotin synthesis genes resulted in full growth of *Rhizobium etli* CFN42 and *Rhizobium phaseoli* CIAT652 strains. Half of rhizobial genomes did not show genes for biotin biosynthesis, but three-quarters had genes for biotin transport. Some strains had genes for an avidin homologue (rhizavidin), a protein with high affinity for biotin but an unknown role in bacteria. A CFN42-derived rhizavidin mutant showed a sharper growth decrease in subcultures, revealing a role in biotin storage. In the search of biotin-independent growth of subcultures, CFN42 and CIAT652 strains with excess aeration showed optimal growth, as they also did, unexpectedly, with the addition of aspartic acid analogues α- and *N*-methyl aspartate. Aspartate analogues can be sensed by the chemotaxis aspartate receptor Tar. A *tar* homologue was identified and its mutants showed no growth recovery with aspartate analogues, indicating requirement of the Tar receptor in such a phenotype. Additionally, *tar* mutants did not recover full growth with excess aeration. A Rubisco-like protein was found to be necessary for growth as the corresponding mutants showed no recovery either with high aeration or aspartate analogues; also, diminished carboxylation was observed. Taken together, our results indicate a route of biotin-independent growth in rhizobial strains that included oxygen, a Tar receptor and a previously uncharacterized Rubisco-like protein.

## Introduction

Rhizobia are alpha proteobacteria that are able to live free in soils and establish symbiotic relationships with legumes. These bacteria induce the formation of nodules on the plant roots and inhabit them to fix atmospheric nitrogen [1]. Strains from several species such as *Rhizobium etli, Rhizobium tropici* and *Sinorhizobium meliloti* show growth reduction when they are serially subcultured in minimal medium with succinate-ammonium [2]. In *R. etli* strain CFN42, this metabolic state included a slow growth rate in the second and third subcultures, excretion of metabolites such as organic acids and amino acids, reduced activity of pyruvate dehydrogenase (PDH) and pyruvate carboxylase (PYC), and accumulation of the reserve polymer poly β-hydroxybutyrate (PHB).

Carbon assimilation was diminished under these conditions, mainly due to the lack of the vitamin biotin, which is the prosthetic group of several carboxylases such as acetyl-CoA carboxylase, propionyl-CoA carboxylase, 3-methyl crotonyl-CoA carboxylase, geranyl-CoA carboxylase, urea carboxylase and PYC. The last enzyme is crucial for anaplerotic carbon assimilation through oxaloacetate formation [3, 4]. These enzymes consist of biotin carboxylase, carboxyltransferase and biotin carboxyl carrier protein components. They are widely distributed in nature and have important functions in carbohydrate assimilation, fatty acid metabolism, urea utilization, polyketide biosynthesis and other processes [5]. Even though biotin is an essential cofactor for the functioning of metabolic carboxylases that fix carbon by incorporating carbon dioxide, many rhizobial strains are biotin auxotrophs, unable to biosynthesize the vitamin [2].

Given the importance of carboxylations and the gradual absence of biotin in rhizobial subcultures, the search for new carboxylation activities is important. Rubisco (ribulose-1,5-bisphosphate carboxylase-oxygenase) is the main carboxylase in nature, and is biotin-independent [6]. However, this carboxylase has not been found in *R. etli *. Rubisco-like protein (RLP) is an ancestral homologue of Rubisco and has been shown to participate in the methionine savage cycle in *Bacillus subtilis,* and in sulphur metabolism and isoprenoid biosynthesis in other bacteria [7–9]. No carboxylase activity has been found for RLP and no functional role has been assigned to this gene in rhizobia.

Excreted metabolites can serve as metabolic signals to modulate the mentioned slow metabolism that occurs in growth-deficient rhizobia. The chemotaxis sensing system is the natural candidate to transduce such signals. Chemotaxis is the directed movement of cells toward assimilable sources of nutrients (or repulsion from damaging compounds). Sensing occurs through a transmembranal system of methyl-accepting proteins (MCPs) or receptors. Receptors transduce the signal to switch the orientation (* Escherichia coli *) [10] or speed (rhizobia) [11] of the flagellum. Four receptors have been best characterized in *E. coli,* responding to compounds such as sugars (*trg*, taxis to ribose and galactose), amino acids (*tar* to aspartate; *tsr* to serine) and dipeptides [10, 12]. An additional sensor responds to oxygen (*aer*). MCPs are methylated by CheB, in response to the sensing of chemoattractants, and this promotes the phosphorylation of CheA [10]. MCP receptors in rhizobia were detected and characterized several years ago [13, 14]. It has been reported that chemotaxis is not essential for rhizobial symbiosis [14], but some mutants showed a delay in nodulation [15]. In *Rhizobium leguminosarum* there are two gene clusters for chemotaxis, including genes for the regulatory cascade of signal transduction and *mcp* genes [16].

We searched for new biotin-independent carboxylations that reactivate the growth of subcultures of *R. etli* and *Rhizobium phaseoli* strains. We found a new pathway in which oxygen, an aspartate Tar receptor and a carboxylase related to Rubisco participated.

## Methods

### Bacterial strains and growth conditions

The bacterial strains used in this work are listed in Table 1. Strains were maintained in rich PY medium. Minimal medium MM contained 1.2 mM K_2_HPO_4_, 0.8 mM MgSO_4_, 0.0184 mM FeCl_3_ and 1.5 mM CaCl_2_ [17]. The nitrogen source was 10 mM NH_4_Cl, and the carbon source was 10 mM succinic acid. Serial subcultures were performed as reported previously [2]. Briefly, flasks with liquid MM were inoculated at an OD_540_ of 0.05 from ovenight PY liquid strain cultures and agitated at 200 rpm at 30 °C. After 24 h, the procedure was repeated to incubate the second subculture, with previous wash of cells with MM, centrifugation and resuspension. The third subculture was incubated similarly, after other 24 h of growth. Growth was determined by measuring the OD_540_ and by protein content, determined by the Lowry assay. Aspartic acid analogues, α-methyl aspartate and *N*-methyl aspartate, were purchased from Sigma and used at 1 mM. The MM was pH balanced after addition of the analogues or aspartic acid. Three experiments of each set of strains were performed. PHB concentration was assessed as described previously [2].

**Table 1.** Strains and plasmids used in this study

Strain or plasmid	Relevant characteristics	Reference
*** Rhizobium etli ***		
CFN42^T^	Wild-type strain, isolated from common bean nodules, Mexico.	Noel *et al.*, 1984 [41]
tar-5	CFN42 derived strain, *tar* mutant. Spr	This work
ravA-1	CFN42 derived strain, rhizavidin mutant. Spr	This work
C21-2	CFN42 derived strain, *rlp* mutant. Spr	This work
*** Rhizobium phaseoli ***		
CIAT652	Wild-type strain, isolated from common bean nodules, Colombia.	González *et al.*, 2010 [26]
6-tar	CIAT652 derived strain, *tar* mutant. Spr	This work
63N1	CIAT652 derived strain, *rlp* mutant. Spr	This work
*** Escherichia coli ***		
Mach1	Host for plasmids	Invitrogen
BL21(DE3)	Expression host for proteins	Invitrogen
**Plasmids**		
pCR2.1 Topo	Cloning vector for PCR products, Ap^r^ Km^r^	Invitrogen
pJQ200SK+	Suicide cloning vector, Gm^r^	Quandt and Hynes, 1993 [42]
pBBR1MCS-5	Broad host-range cloning vector	Kovach *et al.*, 1995 [43]
pMS102loxSp17	Source of the *loxP* Sp cassette, Sp^r^	Martínez-Salazar and Romero, 2000 [22]
pRK2013	Conjugation helper plasmid, Km^r^	Figurski and Helinski, 1979 [44]
pK*mobsacB	Suicide cloning vector, sucrose selection, Km^r^	Schafer *et al.*, 1994 [21]
pJET1.2/blunt	Cloning vector for PCR products, Ap^r^	ThermoScientific
pTopo-ravA600	pCR2.1 Topo containing the PCR amplified rhizavidin gene and flanking nucleotides, Km^r^	This work
pTopo-ravA-ORF	pCR2.1 Topo containing the rhizavidin gene, Km^r^	This work
pBBMCS5-ravA-ORF	pBBR1MCS5 containing the rhizavidin gene	This work
pJQ200-ravA600	pJQ200 containing the PCR-amplified rhizavidin gene and flanking nucleotides, Gm^r^	This work
pJQ200-ravA::loxPSp	pJQ200 containing rhizavidin gene with *loxP* Sp inserted into its *Sal*I site, Gm^r^ Sp^r^	This work
pTarRet	pK*mobsacB containing *tar* gene from CFN42 interrupted by loxP Sp cassette, Sp^r^	This work
pTarCIAT	pK*mobsacB containing *tar* gene from CIAT652 interrupted by loxP Sp cassette, Sp^r^	This work
pRLP652	pK*mobsacB containing *rlp* gene from CIAT652 interrupted by loxPSp cassette, Sp^r^	This work

### Determination of total carbon fixation

Subcultures of strains were assessed for carbon fixation through assimilation of radioactive sodium bicarbonate, ^14^C-NaHCO_3_. A 10 ml aliquot of a second liquid subculture of strains was taken at 8 h and incubated for an additional 40 min with 10 µl ^14^C-NaHCO_3_. Then, the samples were centrifugated and the pellets were resuspended with 1 ml of perchloric acid for quenching. After sitting overnight, samples were combined with 30 ml scintillation fluid (Sigma) and shaken vigorously for 60 min. Incorporation of radioactive carbon was measured with an LS6000 C scintillation counter (Beckman Instruments). Three experiments were performed. Protein content was calculated from the subculture by the Lowry method.

### Chemotaxis experiment

Soft agar plates (0.2 %) with PY rich medium or minimal medium (succinic acid-ammonium) complemented with attractants at 0.1 or 1 mM were spot inoculated with wild-types or *tar* mutant strains. After incubation for 72–96 h, the plates were observed and the cellular dispersion measured.

### Greenhouse assays

Bean seeds of *Phaseolus vulgaris* cultivar Negro Jamapa were germinated and inoculated with 1 ml of bacterial suspensions at an OD_540_ of 0.05, as described previously [18]. Plants were taken for analysis at 12, 17 and 24 days post-inoculation (dpi). The number of nodules, fresh and dry nodule weights, and fresh and dry plant weights were recorded. Nitrogenase activity was determined by the acetylene reduction assay. Specific activity of nitrogenase was calculated by dividing by the dry nodule weight per plant. Significant differences were calculated using Student’s *t*-test. Three experiments were performed. Nodulation competitivity was assessed by co-inoculating mixtures of strains, in wild-type/mutant ratios of 1 : 1, 1 : 10 and 10 : 1. All nodules of 18 dpi plants (*n*=5) were collected; nodule occupancy was discriminated based on the spectinomycin resistance of mutant strains.

### PCR amplification and Southern hybridization

A list of the primers used is given in Table S1 (available in the online version of this article). Specific PCR primers were designed using Primer3 (available at http://frodo.wi.mit.edu) and Oligo v.7 (https://www.oligo.net) programs and were synthesized by the Unidad de Síntesis Química, IBt-UNAM. PCR amplifications were done in a Veriti 96-well Thermal Cycler (Applied Biosystems). Accuprime high-fidelity Taq polymerase (ThermoFisher Scientific) or High Fidelity Pol DNA Polymerase (Jena Bioscience) were used in PCRs with a cycling regime that included a denaturing step at 95 °C for 1 min followed by 30 cycles of 95 °C for 30 s, from 56 to 64 °C (according the primer melting temperature) for 30 s and 72 °C for 90 s. A final elongation step was made at 72 °C for 5–10 min, according to the length of the DNA fragment to be amplified. Southern hybridizations were performed following standard protocols.

### Construction of plasmids and *Rhizobium-*derived strains

For insertional inactivation of the CFN42 rhizavidin gene (RHE_RS29585) [19], genomic DNA was used as template with primers ravA600-F and ravA600-R. The rhizavidin coding region with approximately 600 nt on either side ws amplified by PCR and the amplified region (1.7 kb) was cloned into pCR2.1Topo (Invitrogen) to create plasmid pTopo-ravA600. The rhizavidin gene with flanking regions was excised using the *Spe*I and *Xho*I sites of the vector and the fragment was cloned into pJQ200SK+, cut with the same enzymes, to generate plasmid pJQ200-ravA600. The Sp^r^ element *Sal*I fragment from plasmid pMS102loxSp17 was inserted into the unique *Sal*I site of the gene in pJQ200-ravA600 to generate plasmid pJQ200ravA::loxPSp.

For complementation of the rhizavidin mutant, the rhizavidin coding region including 54 nt upstream of the start codon and 46 nt downstream of the stop codon was amplified by PCR using pTopo-ravA600 as template and primers ravA-F and ravA-R (Table S1). The amplified product (0.6 kb) was cloned into pCR2.1Topo (Invitrogen) to generate pTopo-ravA-ORF. *Hin*dIII and *Xho*I sites of the vector were used to excise the insert and ligate it into the broad-host-range plasmid pBBR1MCS-5 cut with the same enzymes to give pBBMCS5-ravA-ORF, which contains the rhizavidin gene in the same orientation as the *lac* promoter present in the vector. Plasmids pBBMCS5-ravA-ORF or pBBR1MCS-5 were separately transferred into the *R. etli* wild-type or rhizavidin mutant ravA-1 by triparental mating.

*tar* gene sequences from CFN42 (RHE-RS17990) and CIAT652 (RHECIAT-RS18420) strains were amplified and cloned, and a *loxP*Sp cassette was inserted in genetic constructions to obtain mutant strains. In detail, fragments upstream and downstream of both genes were amplified using the primer pairs F1-tarRet – R1-tarRet (upstream) and F2-tarRet – R2-tarRet (downstream) for CFN42 and primer pairs F1-tarCIAT – R1-tarCIAT (upstream) and F2-tarCIAT – R2-tarCIAT (downstream) for CIAT652 (Table S1). The two resulting products were then fused using the overlapping extension PCR methodology [20]. The assembly products were then recovered using the forward primer of the upstream fragment and the reverse primer of the downstream fragment and cloned into the pJET1.2/blunt cloning vector (Thermo Scientific), and sequenced to confirm that the fused product was correctly obtained with no nucleotide changes. These plasmids were digested with *Bgl*II and the fragments of interest were cloned into the suicide plasmid pK*mobsacB [21], digested with *Bam*HI, yielding plasmids pK*mobsacB::tarCFN42 and pK*mobsacB::tarCIAT. These plasmids were further modified by inserting the *loxP*Sp cassette [22] into the unique *Bam*HI site of the constructions, generating plasmids pTarRet and pTarCIAT, respectively.

To introduce a mutation into the *rlp* genes of strains CFN42 (RHE_RS26630) and CIAT652 (RHECIAT_RS30625), a 1578 bp product was obtained by PCR using total DNA from strain CIAT652 and the specific primers F-rlp652 and R-rlp652. The PCR product was cloned by T-A annealing into PCR2.1 TOPO (Invitrogene) and analysed by sequencing. The 1482 bp *Bgl*II*–Bam*HI containing the *rlp* gene was cloned into the suicide plasmid pK*mobsacB [21], and digested with *Bam*HI, generating plasmid pK*mobsacB::rlpCIAT. Mutagenesis of the gene was done by introducing into the unique *Xho*I site of the gene the *loxP*Sp *Sal*I fragment from plasmid pMS102loxSp17 [22], giving plasmid pRLP-652. Both derived mutants were obtained with this plasmid.

Replacement of wild-type alleles (rhizavidin, *tar* and *rlp* in CFN42 and *tar* and *rlp* in CIAT652) by the mutant alleles in plasmids pJQ200ravA::*loxP*Sp, pTarRet, pTarCIAT and pRLP-652, respectively, was carried out by homogenization using the *sacB* marker present in the donor plasmids. Double recombinants were selected as Sac^r^, Sm^r^, Sp^r^, Gm^s^ or Sac^r^, Fos^r^, Km^s^ transconjugants. Replacement was confirmed by Southern hybridization. The profiles of native plasmids were revised by the Eckhardt technique, as modificed by Hynes and McGregor [23]. For biotin biosynthesis complementation, p996 plasmid [24] was mobilized into strain CFN42 through triparenal mating, using pRK2013 as helper.

### Detection of genes for biosynthesis and transport of biotin, and rhizavidins

The complete genomes of 181 strains from *Rhizobiales* were recovered from NCBI (www.ncbi.nlm.nih.gov/genomes) through ftp. Products of genes for biosynthesis (BioABDF) and transport (BioMNY) of biotin were searched using as reference those from *Sinorhizobium fredii* NGR234 with Fasta36 and the following parameters: 30 % identity, 70 % overlap and E score of at least 1e^−5^. Genes coding for avidin homologues, a protein that strongly binds biotin, were searched using that from *R. etli* CFN42, WP_004674376.1. A phylogenetic tree was obtained with PhyML (www.atgc-montpellier.fr/PhyML) using *recA* gene sequences from the rhizobial strains. A representative strain, from each genus, was chosen to mark in the tree the presence or absence of genes for biosynthesis and transport of biotin, and rhizavidin.

### Search of MCP gene homologues and structural comparison

Genes annotated as MCPs in the genomes of strains CFN42 and CIAT652 were recovered. Structural motifs were predicted using InterPro (www.ebi.ac.uk/interpro). Families of MCPs were defined as sharing the sensor motif. MCPs from *S. meliloti* 1021 [13] were used to assign some of them.

## Results and discussion

### *R. etli* and *R. phaseoli* strains require biotin for optimal growth

**Fig. 1.**
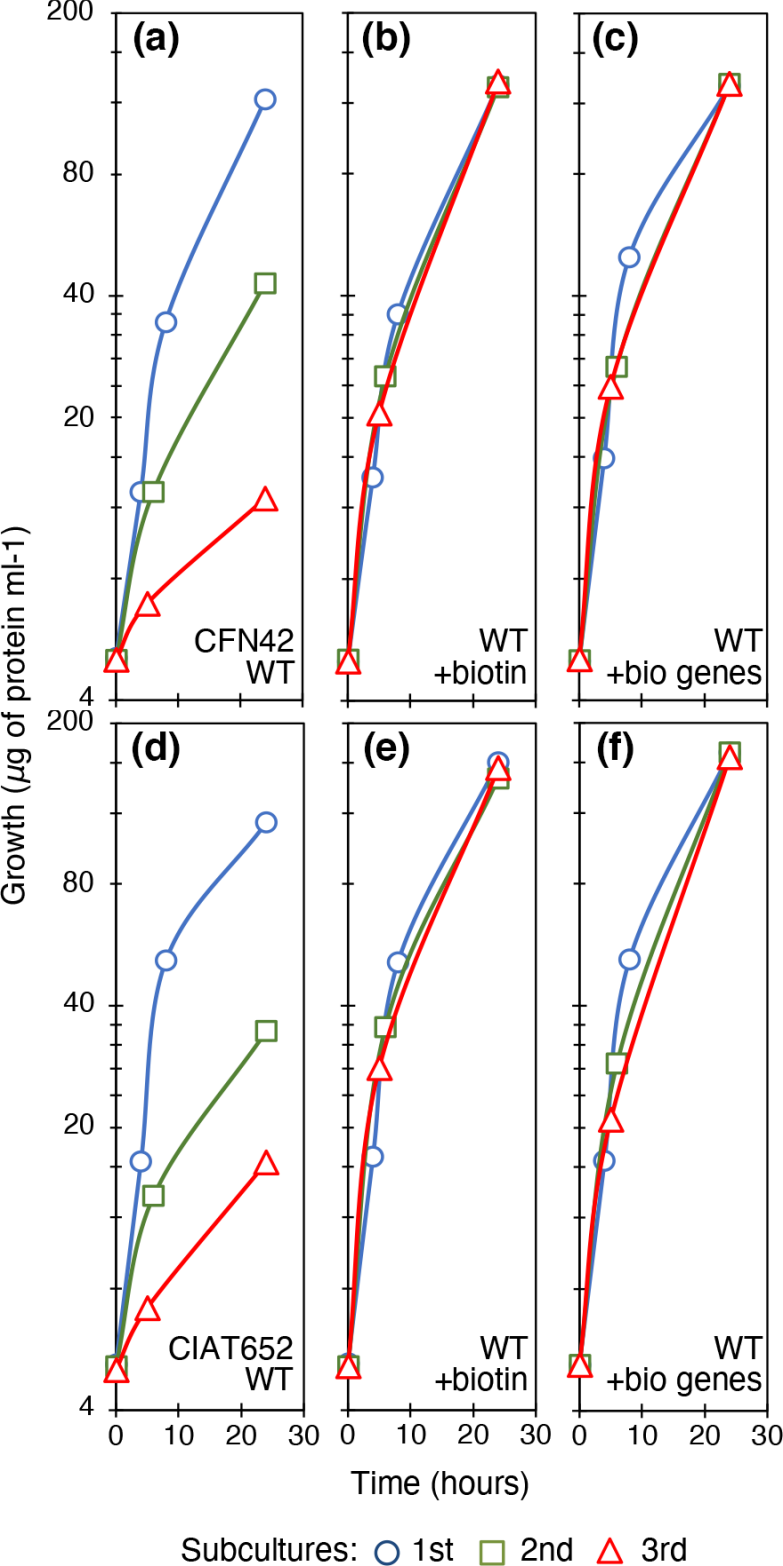
Growth of rhizobial strains in serial cultures. Liquid minimal medium supplemented with succinic acid (10 mM) and ammonium chloride (10 mM). (a) *R. etli* strain CFN42^T^. (b) CFN42 with 1 mM biotin. (c) CFN42 containing plasmid p996 carrying *M. loti bioBDAFZ* genes for the biosynthesis of biotin. (d) *R. phaseoli* strain CIAT652. (e) CIAT652 with 1 mM biotin. (f) CIAT652 containing plasmid p996 carrying *M. loti bioBDAFZ* genes for the biosynthesis of biotin. Line colours and symbols: blue (circles), first subculture; green (squares), second subculture; red (triangles), third subculture. Averages of three experiments are shown. Standard deviations were <5 %.

In this work we have characterized and compared two strains: *R. etli* CFN42^T^, a strain isolated in central Mexico that has been studied for many years in our Centre [25], and *R. phaseoli* CIAT652, from Colombia, a strain used as inoculant in Central America and with good ability to fix nitrogen [26]. Both strains are common bean (*Phaseolus vulgaris*) symbionts. Serial subcultivation in minimal medium succinate-ammonium has been our main approach to determine the growth deficiencies of rhizobia. It is important to note that viable cell numbers correlate well with an increase in cell protein content [2]. In the first culture, the strains grew well, possibly because they had sufficient intracellular amounts of vitamins carried over from the rich medium used to obtain the inoculum. In the second culture, the strains grew less than a half as well as in the first culture, and in the third culture the cells barely reached a tenth of the initial growth (Fig. 1a, d, for CFN42 and CIAT652, respectively). Biotin was a limiting factor for the growth of these subcultures in MM, since full growth recovery was obtained by the addition of 1 mM biotin (Fig. 1b, e, respectively).

To confirm that rhizobia can recover full growth by producing biotin, we transferred the p996 plasmid containing the *bioBFDAZ* operon from *Mesorhizobium loti* strain R7A [24] into the biotin-auxotrophic strains, CFN42 and CIAT652. As observed, growth recovery was optimal (Fig. 1c, f, respectively). Additionally, the biotin-auxotrophic strain *S. meliloti* 1021, containing the *bioBFDAZ* genes, also grew optimally in subcultures (data not shown).

We published the original observation of growth decay in *R. etli* strain CFN42 in 1995 [2]. In the present work we included *R. phaseoli* CIAT652 [26]. The species *R. etli* and *R. phaseoli* share the macrosymbiont but show several metabolic and symbiotic differences. CIAT652 has a better ability to fix nitrogen, has a gene for phosphoenol pyruvate carboxylase (PEPC), produces lower levels of PHB and has a higher rate of ATP synthesis (Vargas-Lagunas *et al*., unpublished results). Here, both strains showed a similar phenotype in growth decay and responded well to the addition of biotin or to genetic complementation that allowed biotin biosynthesis.

### Half of rhizobial genomes do not have genes for biotin biosynthesis

**Fig. 2.**
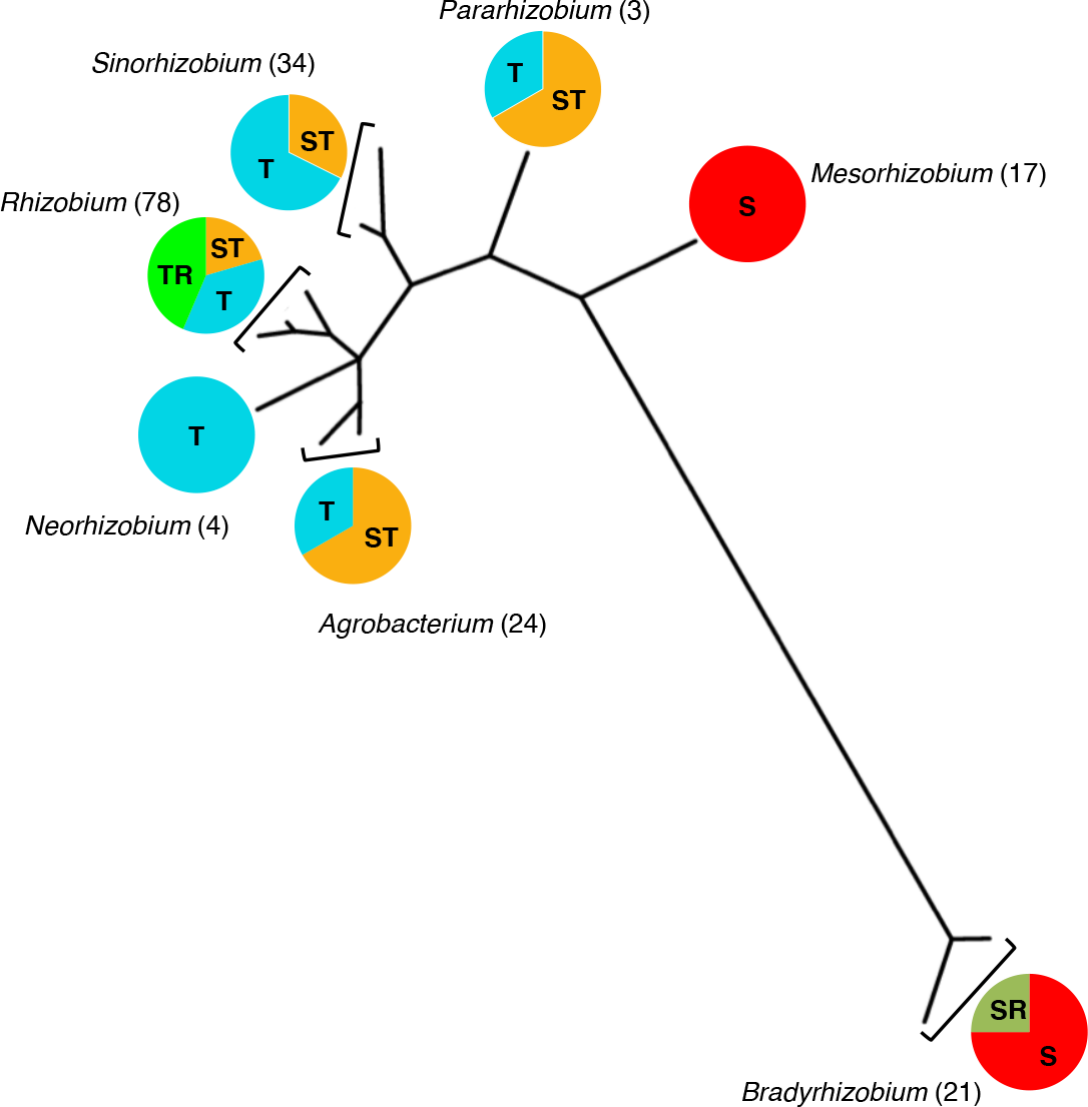
Distribution of genes for biosynthesis and transport of biotin in 181 complete rhizobial genomes. Scheme of the phylogenetic tree obtained using the *recA* gene; only one sequence from each species is shown. The presence of genes for biosynthesis (*bioABDF*) is denoted with S, transport of biotin (*bioMNY*) with T and bradavidin/rhizavidin as R. They are represented by coloured circles and divided by proportions of strains in each genus. Circle colours: red, biosynthesis; orange, biosynthesis and transport; dark green, biosynthesis and bradavidin; blue, transport; light green, transport and rhizavidin. The number of strains is given in parentheses after the genus name.

Given the importance of biotin, we evaluated the occurrence of biotin auxotrophy in rhizobia. Genes for biotin synthesis (presence of *bioABDF* genes) or transport (*bioMNY*) were searched for in the genomes of 181 rhizobial strains in seven genera (*Bradyrhizobium, Mesorhizobium, Pararhizobium, Neorhizobium, Rhizobium, Agrobacterium* and *Sinorhizobium *). Of these strains, 98 (54 %) had genes for transport, 38 (21 %) had genes for biosynthesis, and 45 (24.8 %) had genes for both capabilities (Figs 2 and S1). Apparently, the presence or absence of genes for these features followed a phylogenetic trend. For example, only the *Rhizobiaceae* showed genes for biotin transport, and the genera *Bradyrhizobium* and *Mesorhizobium* presented biotin biosynthesis only. Looking for other genes interacting with biotin, we searched for avidin homologues. The first bacterial homologue of avidin was found in *Bradyrhizobium japonicum* USDA110 and named bradavidin [27]. Rhizavidin was isolated later in *R. etli* CFN42 [28]. We found that avidin homologues were present in some strains of *Bradyrhizobium* (co-occurring with genes for biotin biosynthesis) and *Rhizobium* (co-occurring with genes for biotin transport). No specific role has been assigned to avidins in bacteria. We propose that rhizavidin participates in the cellular strategy to capture and conserve biotin.

This analysis on biotin auxotrophy in rhizobia is the most complete performed to date. Previous studies on the presence of biotin synthesis and transport were limited and used very few strains from some species [29]. We analysed all available rhizobial genomes (around 500), but many were unfinished and were thus discarded because they did not allow a precise analysis. The remaining 181 genomes were sufficient to provide sufficient scope to reveal interesting phylogenetic tendencies. The evolutionary significance of vitamin auxotrophy has been studied. It appears to be related to the environment in which bacteria develop. If there is an abundance of a particular vitamin, the microorganism eventually will lose the biosynthetic genes, as has been observed with cobalamin auxotrophy during experimental evolution in *Chlamydomonas reinhardtii* [30]. In the case of biotin and rhizobia, the vitamin-containing environment could be the legume root nodule. Thus, it is possible that the preferred lifestyle of rhizobia, nitrogen-fixing symbiosis, caused widespread auxotrophy in the family. In general, as observed in the genomes of obligate endosymbionts or parasites, the loss of genetic material for the synthesis of diverse building block compounds is thought to be evolutionarily advantageous [31].

### Rhizavidin has a role in biotin conservation

**Fig. 3.**
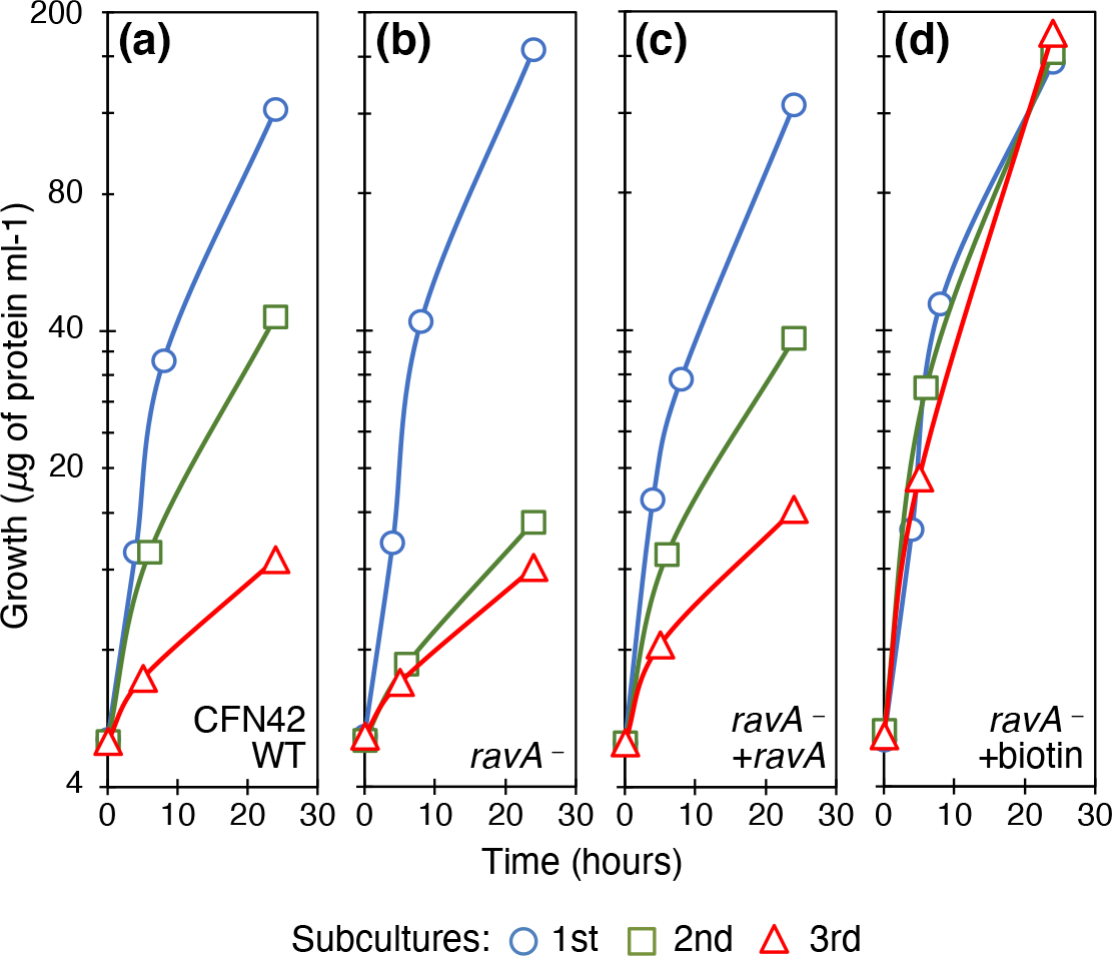
Characterization of *R. etli* CFN42-derived rhizavidin mutant strain. Growth of strains in serial cultures in MM of succinate-ammonium. (a) *R. etli* CFN42 wild-type strain. (b) ravA-1 mutant strain. (c) ravA-1 mutant strain complemented with the rhizavidin gene in a broad-host-range plasmid, pBBR1MCS5. (d) ravA-1 mutant strain with 1 mM biotin. Line colours and symbols: blue (circles), first subculture; green (squares), second subculture; red (triangles), third subculture. Averages of three experiments are shown. Standard deviations were <5 %.

To explore the role of rhizavidin in metabolism, we identified the gene coding for this protein in *R. etli* CFN42 and a mutant was obtained. In this strain, named ravA-1, we found increased inhibition of aerobic metabolism, showing faster growth decay in the second subculture and null growth in the third, in comparison with the wild-type strain (Fig. 3a, b). Complementation of the mutant strain restored the wild-type phenotype (Fig. 3c). The addition of biotin corrected the mutant phenotype and produced optimal growth (Fig. 3d). Thus, the protein rhizavidin was essential to conserve and store biotin. Furthermore, we determined the PHB accumulation of the strains in subcultures because such a phenotype suggests biotin deficiency. The rhizavidin rav-1 mutant accumulated six times more PHB (4.5±0.1 mg PHB/mg protein) than the wild-type strain (0.8±0.1 mg of PHB/mg of protein) in the second subculture. Such accumulation correlated with more deficient growth, as has been observed previously [2]. The addition of biotin strongly reduced PHB accumulation to basal levels in the rav-1 mutant (0.1 mg PHB/mg protein), as was also found in the wild-type strain (0.1 mg PHB/mg protein).

Avidin was first discovered and isolated from chicken egg white. Biotin is an important factor for embryo development. The precise function of avidins has not been elucidated, with proposals ranging from biotin transport to protection by sequestering biotin to avoid microbial growth [32]. Up to 50 % of biotin consumption by hens is deposited in the eggs. The high affinity of avidin for biotin is so strong that this characteristic has converted the pair into a widely used tool in protein science and biotechnology [33]. The non-covalent association (*K*_m_) of biotin with avidin is in the order of 10^−15^ M, with a very slow dissociation rate. The first bacterial homologue of avidin was discovered in the rhizobia. We wondered about the role of avidin homologue in biotin metabolism. The function found here of biotin storage and conservation accorded well expectations regarding the importance of the vitamin in the metabolism of rhizobia, and discarded the possibility of avidins as biotin sequesters, as has seen for example in toxicity for mice in maize [34]. We propose that rhizavidin’s role is to conserve biotin and make it available for carboxylation reactions. This was confirmed in our experiments, as the absence of rhizavidin exacerbated the growth reduction phenotype.

### Non-metabolizable aspartate analogues and oxygen helped to recover total growth

**Fig. 4.**
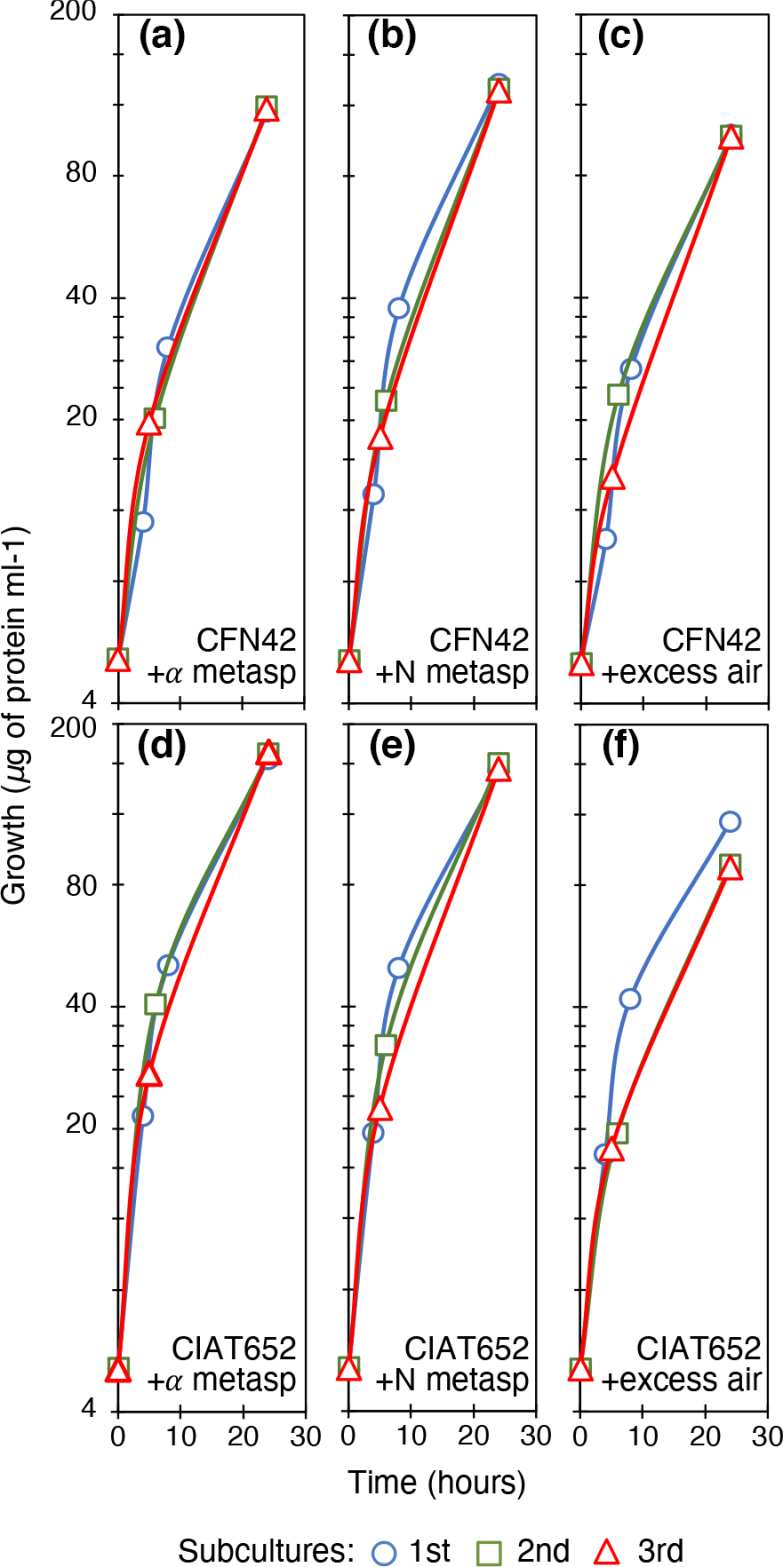
Effect of aspartic acid analogues and excess air on growth of strains. Subcultures of MM of succinate-ammonium. (a) *R. etli* strain CFN42 with 1 mM α-methyl aspartic acid. (b) CFN42 with 1 mM *N*-methyl aspartic acid. (c) CFN42 bubbled with excess air. (d) *R. phaseoli* strain CIAT652 with 1 mM α-methyl aspartic acid. (e) CIAT652 with 1 mM *N*-methyl aspartic acid. (f) CIAT652 bubbled with excess air. Line colours and symbols: blue (circles), first subculture; green (squares), second subculture; red (triangles), third subculture. Averages of three experiments are shown. Standard deviations were <5 %.

Given the absence of biotin synthesis in the subcultures and the importance of carboxylation, we searched for biotin-independent growth in the rhizobial strains. We found that some metabolites were able to promote optimal growth, such as malate plus fumarate, and thiamine (data not shown). Strikingly, two structural analogues of aspartic acid, namely α-methyl aspartate (Fig. 4a, d, for CFN42 and CIAT652, respectively) and *N*-methyl aspartate (Fig. 4b, e, respectively) promoted full growth of strains CFN42 and CIAT652. These analogues cannot be used as C and/or N sources (data not shown). Aspartic acid did not have this effect when tested at 1 or 10 mM (Fig. S2). Also, we found that when compressed air was bubbled into the subcultures, growth recovery was complete (Fig. 4c, f, for CFN42 and CIAT652, respectively).

From the first studies on chemotaxis, structural analogues have played a significant role [35]. Some advantages are the inability for metabolization, conserving a strong chemotactic response and maintaining the study concentration. The analogues helped to demonstrate that chemoattraction to the substances can be independent of the metabolic benefit of transport or can metabolize them [36]. We consider that the aspartate analogues interact with the dedicated MCP receptor and in this way a signal is sent in such a manner that it activates metabolism. Aspartic acid did not stimulate optimal growth, because it can be assimilated and thus eliminates the activation signal.

### Genomic search of *tar* homologues in strains CFN42 and CIAT652

The system that senses aspartic acid and its analogues might be the chemotaxis sensing system, through MCPs in the membrane, specifically by the aspartate receptor [31]. Consequently, we decided to study the role of the *tar* receptor in the metabolism.

**Fig. 5.**
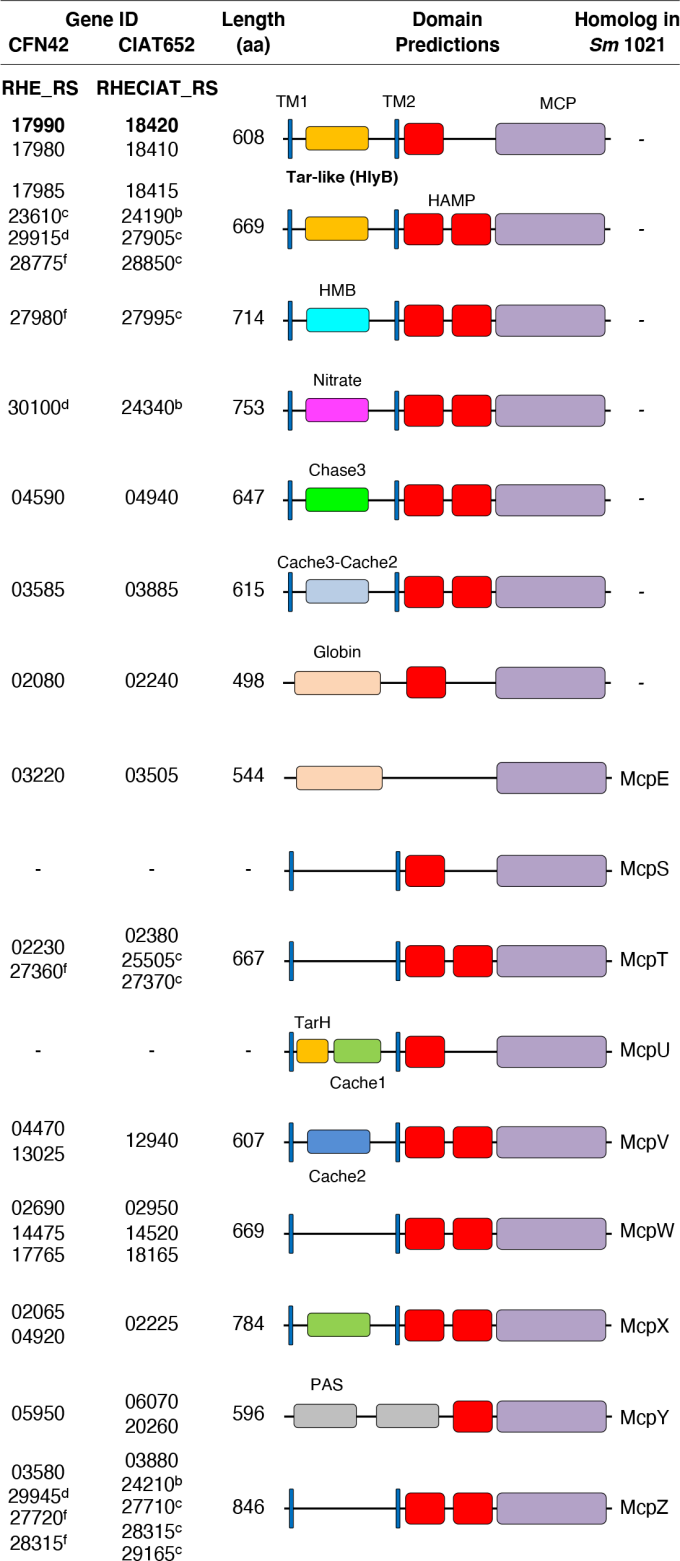
Schematic representation of MCP chemoreceptors identified in the genomes of *R. etli* CFN42 and *R. phaseoli* CIAT652. MCPs were detected using InterPro searches. *S. meliloti* strain 1021 MCP designations are from Meier *et al*. [13]. Blue bars, TM1 and TM2 transmembranal segments. HAMP domains, red boxes. MCP domains, violet boxes. MCPs with HlyB receptor domain were proposed as the *tar* family (aspartic acid-responding). In bold are genes proposed as *tar*. A letter in gene IDs denotes plasmid location while no letter denotes location in the chromosome.

In the search for *tar* homologues in *R. etli* CFN42 and *R. phaseoli* CIAT652, we performed a detailed genomic analysis for the presence and structure of MCP receptors (Fig. 5). We identified 26 and 27 MCP homologues in CFN42 and CIAT652, respectively, with diverse degrees of conservation in comparison with the best characterized receptors, i.e. those from *E. coli* [35]. The proposed family of *tar* homologues in our strains contained an HlyB sensing motif and had six genes in each strain; two of them (genes RHE_RS17985-RHE_RS17990 in CFN42, and RHECIAT_RS18415-RHECIAT_RS18420 in CIAT652, respectively) were contiguous, within the *che2* cluster. Both MCPs in the *che2* cluster had the highest homology to the *E. coli tar,* showing the same conserved structure. Thus, RHE_RS17990 and RHECIAT_RS18420 genes were designated as *tar* genes.

The abundance of receptors is a characteristic shared with other proteobacteria living in a complex environment such as soil [37]. For example, *Agrobacterium tumefaciens* C58 was found to present 22 MCP receptors [38].

### The *tar* mutants did not recover full growth in minimal medium

The *tar* mutants were obtained through deletion of the respective gene. The mutants were named tar-5 (derived from CFN42) and 6-tar (derived from CIAT652). The mutant strains were characterized by motility, growth and symbiotic ability. The *tar* mutants showed reduced motility in rich medium PY and on MM plates, especially with aspartic acid, succinate, and malate added (Fig. S3). In general, the effect was stronger in mutant 6-tar.

**Fig. 6.**
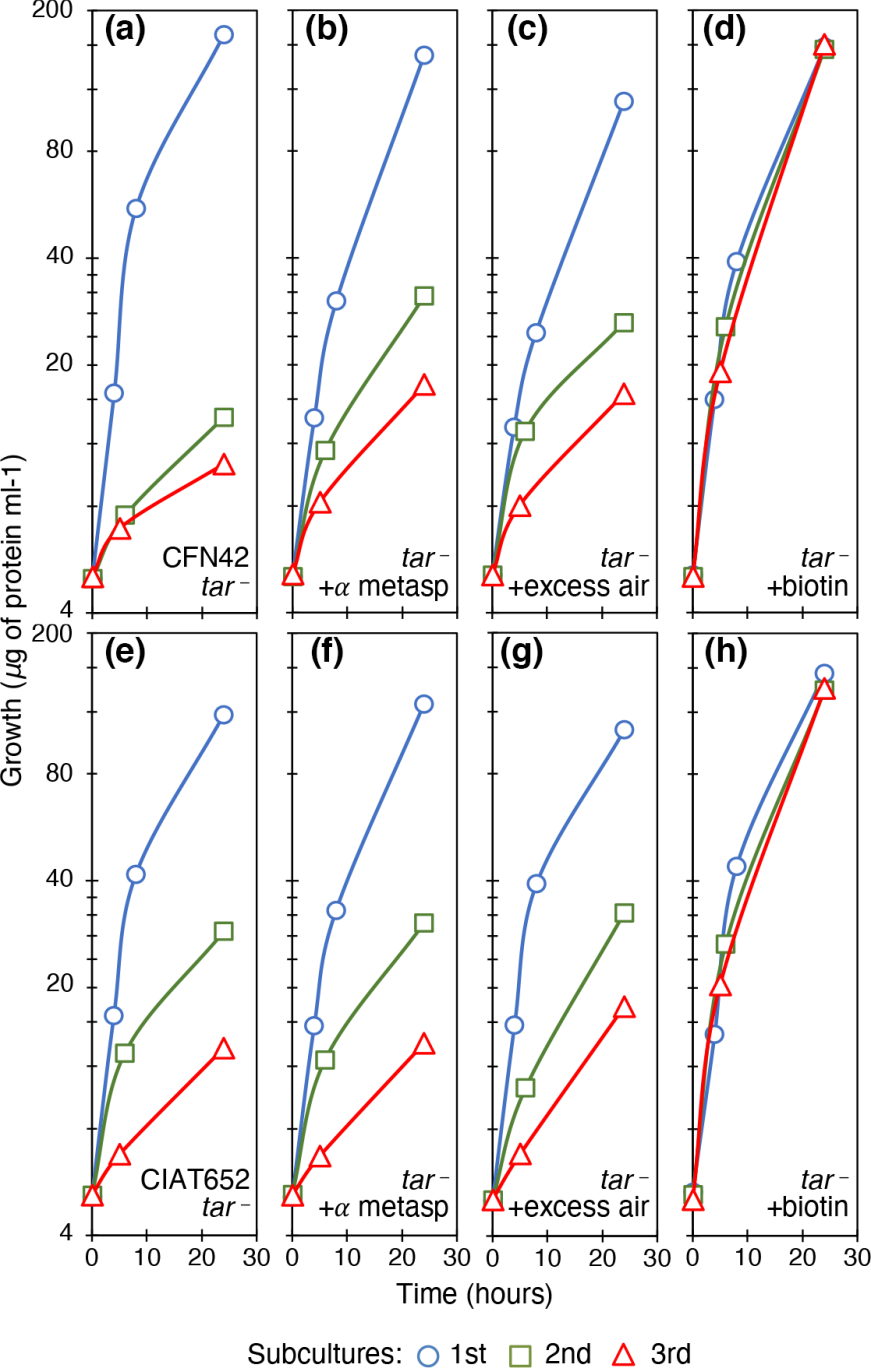
Characterization of *R. etli* CFN42- and *R. phaseoli* CIAT652-derived *tar* mutant strains. Subcultures in MM of succinate-ammonium. (a) The tar-5 mutant strain, derived from CFN42. (b) The tar-5 mutant strain with 1 mM α-methyl aspartic acid. (c) The tar-5 mutant strain bubbled with excess air. (d) The tar-5 mutant strain with 1 mM biotin. (e) The 6-tar mutant strain, derived from CIAT652. (f) The 6-tar mutant strain with 1 mM α-methyl aspartic acid. (g) The 6-tar mutant strain bubbled with excess air. (h) The 6-tar mutant strain with 1 mM biotin. Line colours and symbols: blue (circles), first subculture; green (squares), second subculture; red (triangles), third subculture. Averages of three experiments are shown. Standard deviations were <5 %.

When compared with the wild-type strain CFN42 (see Fig. 1a for reference), the *tar* mutant strain unexpectedly showed significant growth reduction, in the second and the third subcultures in MM (Fig. 6a). This indicated that *tar* mutants had an effect on metabolism not described before. In the case of 6-tar, the CIAT652-derived mutant strain, no change was apparent in the subcultures (Fig. 6e). Furthermore, the full growth observed in the wild-type strains by the addition of the aspartate analogue α-methyl aspartate, did not occur in the *tar* mutants (Fig. 6b, f, for the tar-5 and 6-tar strains, respectively). Thus, the *tar* deletion blocked the ability to respond to the analogue. Also, the full-growth phenotype of the wild-type strains observed with excess air was not obtained in serial cultures of *tar* mutants (Fig. 6c, g, for the tar-5 and 6-tar strains, respectively). The sensing of strong aeration (i.e. oxygen) came via Tar, and also showed that the route is shared with the sensing of aspartate analogues. When biotin was added to the subcultures of *tar* mutants, it allowed full growth (Fig. 6d, h, for the tar-5 and 6-tar strains, respectively).

The metabolic effect of the *tar* mutation was unexpected, given that no similar reports have been found in the literature. Only in *Azospirillum brasilense* has a protein named SbpA been shown to participate in chemotaxis and sugar uptake [17]. It appears that the chemotactic system is not coupled to the assimilation of the compounds. However, our results revealed a more connected function between signal reception and metabolism.

### CIAT652 tar mutant induced a strong negative symbiotic response in common bean plants

**Fig. 7.**
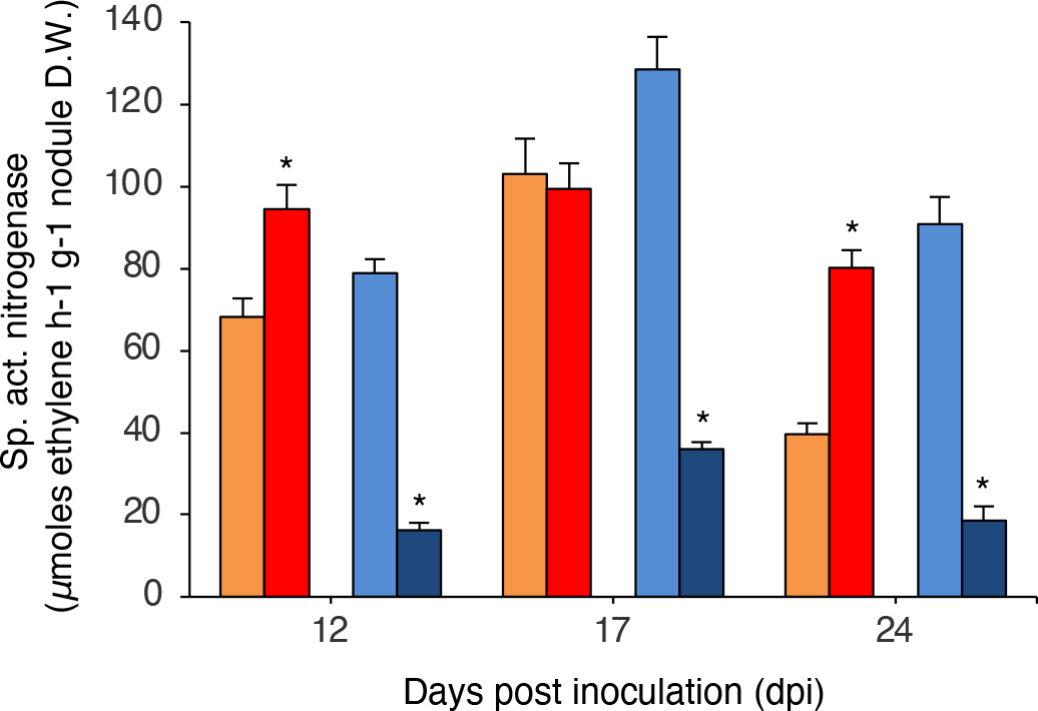
Symbiotic effect of the *tar* mutation. Common bean plants (*Phaseolus vulgaris*) grown in the greenhouse were inoculated with rhizobial strains. Nitrogenase-specific activity was determined at 12, 17 and 24 days post-inoculation; averages of 10 plants per date, with standard deviation, are shown. Asterisks represent significant differences (*P*≤0.05, Student's *t*-test) in comparison with its wild-type strain. A representative of three experiments is shown. Bar colours: orange, *R. etli* CFN42 wild-type strain; red, CFN42-derived tar-5 mutant strain; light blue, *R. phaseoli* CIAT652 wild-type strain; dark blue, CIAT652-derived 6-tar mutant strain.

To gain more insight into the importance of the *tar* receptor, we tested the *tar* mutant strains in symbiosis with *Phaseolus vulgaris* (common bean) plants in the greenhouse. Strikingly, *tar* mutants again had contrasting phenotypes with respect to their wild-type strains. The tar-5 mutant had a similar symbiotic ability regarding nitrogenase activity in comparison with CFN42 (Fig. 7). However, the 6-tar mutant strain had lower nitrogenase activity than its wild-type strain (Fig. 7). We analysed the nodulation formation competitivity of the strains; in a 1 : 1 co-inoculation test against their wild-type strains we found that the tar-5 strain occupied 78.8±8.1 % of nodules (expected 50 %) against CFN42; however, the 6-tar strain occupied only 4±3 % of nodules in competition against CIAT652.

The phenotypic differences observed between the mutant *tar* strains are possibly due to other characteristics such as different transcriptional regulation or genetic complementation by members of the *tar* family.

### A Rubisco-like protein participated in growth recovery

**Fig. 8.**
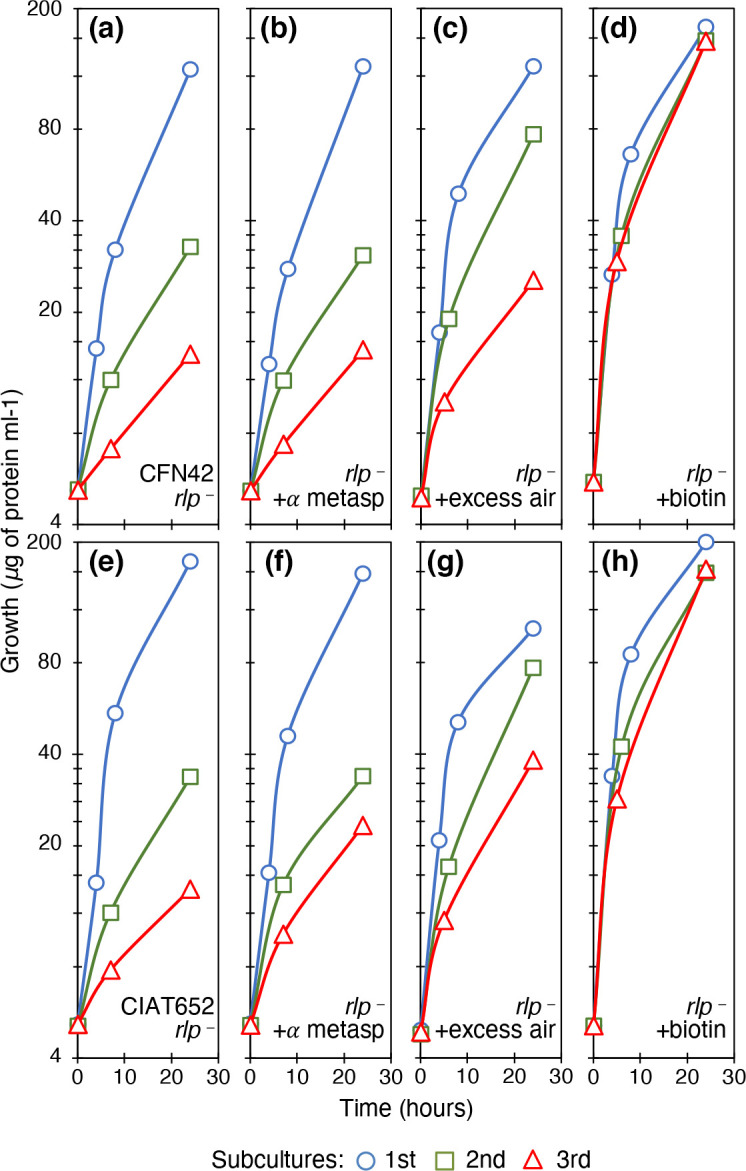
Rubisco-like protein mutant characterization. Subcultures in MM of succinate-ammonium. (a) C21-2 mutant strain, derived from CFN42. (b) C21-2 mutant strain with 1 mM α-methyl aspartic acid. (c) C21-2 mutant strain bubbled with excess air. (d) C21-2 mutant strain with 1 mM biotin. (e) 63N1 mutant strain, derived from CIAT652. (f) 63N1 mutant strain with 1 mM α-methyl aspartic acid. (g) 63N1 mutant strain bubbled with excess air. (h) 63N1 mutant strain with 1 mM biotin. Line colours and symbols: blue (circles), first subculture; green (squares), second subculture; red (triangles), third subculture. Averages of three experiments are shown. Standard deviations were <5 %.

Given the importance of carboxylation for cellular metabolism, and considering that biotin is lacking in the subcultures, we looked for biotin-independent carboxylases in the genomes of rhizobial strains. A homologue of the ribulose *bis*-phosphate carboxylase (Rubisco)-like protein (RLP) in strains CFN42 (RHE_RS26630) and CIAT652 (RHECIAT_RS30265) was found. No carboxylase activity has been found for RLP and no functional role has been assigned in the rhizobia. We obtained mutants of the RLP genes of CFN42 and CIAT652 (C21-2 and 63N1 strains, respectively). These *rlp* mutants grew very similar to their wild-type strains in MM subcultures (Fig. 8a, e, respectively); however, they were unable to obtain full growth in the presence of α-methyl aspartate (Fig. 8b, f, respectively for strains C21-2 and 63N1). According to the proposal that oxygen participates in the same pathway of aspartate analogue sensing for metabolic reactivation, when the strains were tested with excess aeration, the *rlp* mutants did not reach full growth (Fig. 8c, g, respectively). The addition of biotin corrected the phenotype (Fig. 8d, h). To determine whether RLP influences carbon fixation, we assessed total carbon dioxide uptake of strain subcultures, using radioactive sodium bicarbonate (^14^C). We again found differences between the strains. Diminished carbon fixation was observed in the 63N1 *rlp* mutant strain, 1047.3±37.3 c.p.m. μg^−1^ protein compared with its wild-type CIAT652, 630.2±107.2; however, values for CFN42 and C21-2 mutant strain were 1330.1±272.2 and 1045.5±216.6 c.p.m. μg^−1^ protein, respectively. Thus, the participation of Rubisco-like protein in CO_2_ fixation was more evident in the biotin-auxotrophic strain CIAT652.

Rubisco is an essential enzyme for living organisms. It is one of the most abundant proteins on Earth and allows the incorporation of significant amounts of carbon into organic molecules. Four forms of the enzyme have been established, and the first three were shown to be capable of fixing carbon dioxide. The fourth, related to Rubisco and possibly ancestral, showed diverse functions in some bacterial strains [39]. Since its discovery in 1999, form IV or RLP has been studied because it is widely spread in several phyla, but has roles that need to be thoroughly investigated [8]: for example, RLP's role as enolase or tautomerase acting on 4C or 5C substrates in sulphur and methionine metabolism, stress, threonate and erithrose assimilation, isoprenoid metabolism [9], and more recently hamamelose [40]. In *Ochrobatrum anthropi*, a close relative of rhizobia, the homologue *rlp* is surrounded by genes for the assimilation of hamamelose, performing the last step in its conversion to the phosphorylated compound that originates from 3-phosphoglycerate and glycolate [40]. In strains CIAT652 and CFN42, *rlp* neighbours are related to ribose transport and assimilation (data not shown), supporting the possible role of RLP in the carboxylation of a compound such ribulose *bis*-phosphate.

In this study we found a biotin-independent route to achieve full growth in subcultures of biotin-auxotrophic strains. The participants in this alternative route are oxygen, a Tar receptor and a newly characterized carboxylase related to Rubisco. In addition to the classic regulation of motility, the chemotaxis sensing system, through membrane receptors such as *tar*, played a new metabolic role as indicated by the decreased growth of the CFN42 *tar* mutant (Fig. 6) and reduced nitrogen-fixing capability of the CIAT652 *tar* mutant (Fig. 7). More importantly, CFN42 and CIAT652-derived mutants of the Rubisco-like protein were unable to obtain full growth in the presence of aspartate analogues or excess oxygen, and had diminished carboxylation in CIAT652, indicating the participation of this enzyme in CO_2_ fixation (Fig. 8).

## Supplementary Data

Supplementary material 1

## References

[1] Masson-Boivin C, Sachs JL (2018). Symbiotic nitrogen fixation by rhizobia-the roots of a success story. Curr Opin Plant Biol.

[2] Encarnación S, Dunn M, Willms K, Mora J (1995). Fermentative and aerobic metabolism in Rhizobium etli. J Bacteriol.

[3] Dunn MF, Encarnación S, Araíza G, Vargas MC, Dávalos A (1996). Pyruvate carboxylase from Rhizobium etli: mutant characterization, nucleotide sequence, and physiological role. J Bacteriol.

[4] Dunn MF, Araíza G, Mora J (2004). Biochemical characterization of a *Rhizobium etli* monovalent cation-stimulated acyl-coenzyme A carboxylase with a high substrate specificity constant for propionyl-coenzyme A. Microbiology (Reading).

[5] Tong L (2013). Structure and function of biotin-dependent carboxylases. Cell Mol Life Sci.

[6] Yokota A (2017). Revisiting RuBisCO. Biosci Biotechnol Biochem.

[7] Ashida H, Saito Y, Kojima C, Kobayashi K, Ogasawara N (2003). A functional link between RuBisCO-like protein of Bacillus and photosynthetic RuBisCO. Science.

[8] Hanson TE, Tabita FR (2003). Insights into the stress response and sulfur metabolism revealed by proteome analysis of a *Chlorobium tepidum* mutant lacking the Rubisco-like protein. Photosynthesis Research.

[9] Erb TJ, Evans BS, Cho K, Warlick BP, Sriram J (2012). A RubisCO-like protein links SAM metabolism with isoprenoid biosynthesis. Nat Chem Biol.

[10] Bi S, Sourjik V (2018). Stimulus sensing and signal processing in bacterial chemotaxis. Curr Opin Microbiol.

[11] Götz R, Schmitt R (1987). Rhizobium meliloti swims by unidirectional, intermittent rotation of right-handed flagellar helices. J Bacteriol.

[12] Alanis-Sánchez BM, Pérez-Tapia SM, Vázquez-Leyva S, Mejía-Calvo I, Macías-Palacios Z (2019). Utilization of naproxen by *Amycolatopsis* sp. Poz 14 and detection of the enzymes involved in the degradation metabolic pathway. World J Microbiol Biotechnol.

[13] Meier VM, Muschler P, Scharf BE (2007). Functional analysis of nine putative chemoreceptor proteins in *Sinorhizobium meliloti*. J Bacteriol.

[14] Caetano-Anollés G, Wall LG, De Micheli AT, Macchi EM, Bauer WD (1988). Role of motility and chemotaxis in efficiency of nodulation by *Rhizobium meliloti*. Plant Physiol.

[15] Yost CK, Del Bel KL, Quandt J, Hynes MF (2004). *Rhizobium leguminosarum* methyl-accepting chemotaxis protein genes are down-regulated in the pea nodule. Arch Microbiol.

[16] Yost CK, Rochepeau P, Hynes MF (1998). *Rhizobium leguminosarum* contains a group of genes that appear to code for methyl-accepting chemotaxis proteins. Microbiology (Reading).

[17] Van Bastelaere E, Lambrecht M, Vermeiren H, Van Dommelen A, Keijers V (1999). Characterization of a sugar-binding protein from *Azospirillum brasilense* mediating chemotaxis to and uptake of sugars. Mol Microbiol.

[18] Mora Y, Díaz R, Vargas-Lagunas C, Peralta H, Guerrero G (2014). Nitrogen-fixing rhizobial strains isolated from common bean seeds: phylogeny, physiology, and genome analysis. Appl Environ Microbiol.

[19] Helppolainen SH, Nurminen KP, Määttä JAE, Halling KK, Slotte JP (2007). Rhizavidin from *Rhizobium etli*: the first natural dimer in the avidin protein family. Biochem J.

[20] Shevchuk NA, Bryksin AV, Nusinovich YA, Cabello FC, Sutherland M (2004). Construction of long DNA molecules using long PCR-based fusion of several fragments simultaneously. Nucleic Acids Res.

[21] Schäfer A, Tauch A, Jäger W, Kalinowski J, Thierbach G (1994). Small mobilizable multi-purpose cloning vectors derived from the *Escherichia coli* plasmids pK18 and pK19: selection of defined deletions in the chromosome of *Corynebacterium glutamicum*. Gene.

[22] Martinez-Salazar JM, Romero D (2000). Role of the ruvB gene in homologous and homeologous recombination in *Rhizobium etli*. Gene.

[23] Hynes MF, McGregor NF (1990). Two plasmids other than the nodulation plasmid are necessary for formation of nitrogen-fixing nodules by *Rhizobium leguminosarum*. Mol Microbiol.

[24] Sullivan JT, Brown SD, Yocum RR, Ronson CW (2001). The bio operon on the acquired symbiosis island of *Mesorhizobium* sp. strain R7A includes a novel gene involved in pimeloyl-CoA synthesis. Microbiology (Reading).

[25] Quinto C, De La Vega H, Flores M, Leemans J, Cevallos MA (1985). Nitrogenase reductase: A functional multigene family in *Rhizobium phaseoli*. Proc Natl Acad Sci U S A.

[26] González V, Acosta JL, Santamaría RI, Bustos P, Fernández JL (2010). Conserved symbiotic plasmid DNA sequences in the multireplicon pangenomic structure of *Rhizobium etli*. Appl Environ Microbiol.

[27] Nordlund HR, Hytönen VP, Laitinen OH, Kulomaa MS (2005). Novel avidin-like protein from a root nodule symbiotic bacterium, *Bradyrhizobium japonicum*. J Biol Chem.

[28] Meir A, Helppolainen SH, Podoly E, Nordlund HR, Hytönen VP (2009). Crystal structure of rhizavidin: insights into the enigmatic high-affinity interaction of an innate biotin-binding protein dimer. J Mol Biol.

[29] Guillén-Navarro K, Encarnación S, Dunn MF (2005). Biotin biosynthesis, transport and utilization in rhizobia. FEMS Microbiol Lett.

[30] Helliwell KE, Collins S, Kazamia E, Purton S, Wheeler GL (2015). Fundamental shift in vitamin B12 eco-physiology of a model alga demonstrated by experimental evolution. ISME J.

[31] Merhej V, Royer-Carenzi M, Pontarotti P, Raoult D (2009). Massive comparative genomic analysis reveals convergent evolution of specialized bacteria. Biol Direct.

[32] White 3rd HB (1985). Biotin-binding proteins and biotin transport to oocytes. Ann N Y Acad Sci.

[33] Hytonen VP (2020). (Strept)avidin as a template for ligands other than biotin: an overview. Meth Enzymol.

[34] Kramer KJ, Morgan TD, Throne JE, Dowell FE, Bailey M (2000). Transgenic avidin maize is resistant to storage insect pests. Nat Biotechnol.

[35] Adler J (1969). Chemoreceptors in bacteria. Science.

[36] Mesibov R, Adler J (1972). Chemotaxis toward amino acids in *Escherichia coli*. J Bacteriol.

[37] Parales RE, Luu RA, Chen GY, Liu X, Wu V (2013). Pseudomonas putida F1 has multiple chemoreceptors with overlapping specificity for organic acids. Microbiology.

[38] Xu N, Wang M, Yang X, Xu Y, Guo M (2020). In silico analysis of the chemotactic system of *Agrobacterium tumefaciens*. Microb Genom.

[39] Tabita FR, Hanson TE, Li H, Satagopan S, Singh J (2007). Function, structure, and evolution of the RubisCO-like proteins and their RubisCO homologs. Microbiol Mol Biol Rev.

[40] Kim SM, Lim HS, Lee SB (2018). Discovery of a RuBisCO-like protein that functions as an oxygenase in the novel d-hamamelose pathway. Biotechnol Bioproc E.

[41] Noel KD, Sanchez A, Fernandez L, Leemans J, Cevallos MA (1984). *Rhizobium phaseoli* symbiotic mutants with transposon Tn5 insertions. J Bacteriol.

[42] Quandt J, Hynes MF (1993). Versatile suicide vectors which allow direct selection for gene replacement in gram-negative bacteria. Gene.

[43] Kovach ME, Elzer PH, Hill DS, Robertson GT, Farris MA (1995). Four new derivatives of the broad-host-range cloning vector pBBR1MCS, carrying different antibiotic-resistance cassettes. Gene.

[44] Figurski DH, Helinski DR (1979). Replication of an origin-containing derivative of plasmid RK2 dependent on a plasmid function provided in trans. Proc Natl Acad Sci U S A.

