# Supplementary material for: A Tar aspartate receptor and Rubisco-like protein substitute biotin in the growth of rhizobial strains
Source: Microbiology (Reading). 2022 Jan 25;168(1):001130. doi: 10.1099/mic.0.001130 (PMC8914248; doi:10.1099/mic.0.001130)
Supplement: Supplementary material 1 [file mic-168-1130-s001.pdf]

Supplementary Table 1. Primers used in this work.

Plasmid	Primer	Sequence 5’ → 3’
pJQ200-ravA600	ravA600-F	TCATATGGGTGACGTGATGC
	ravA600-R	CGATGTTCAACCTCATACGAC
pBBR1MCS5-ravA-ORF	ravA-F	TATGCAACCTTCGGTACGAT
	ravA-R	TTGCTCCACAATCTCTGACG
pK**mobsacB*::tarCFN42	F1-tarRet	AC*AGATCT*ATCCCGCTTGCGGCCGTTGAGG
	R1-tarRet	GC*GGATCC*TACGGCTTTAACCCCAAG
	F2-tarRet	CTTGGGGTTAAAGCCGTA*GGATCC*GCTTCCCTTG
	R2-tarRet	ACCA*AGATCT*ATGAGGACGGCTATGC
pK**mobsacB*::tarCIAT	F1-tarCIAT	AGGAATGCCTCGAACTGTCACTCGAGGAAG
	R1-tarCIAT	GCGC*GGATCC*TTACGTCAAACCCCCACGTT
	F2-tarCIAT	CGTAA*GGATCC*GCGCTCCCCTGGGGAGA
	R2-tarCIAT	CAGACCGGTGTTGATTCCGAAGGT
pK**mobsacB*::rlpCIAT	F-rlp652	GGTGATC*AAGCTT*CGCTCGAGTCGATC
	R-rlp652	ACGTCGGT*GGATCC*GGTGAAATCATCC

The restriction sites introduced by the primers are in cursive.
Underlined nucleotides indicate the overlap region between primers.

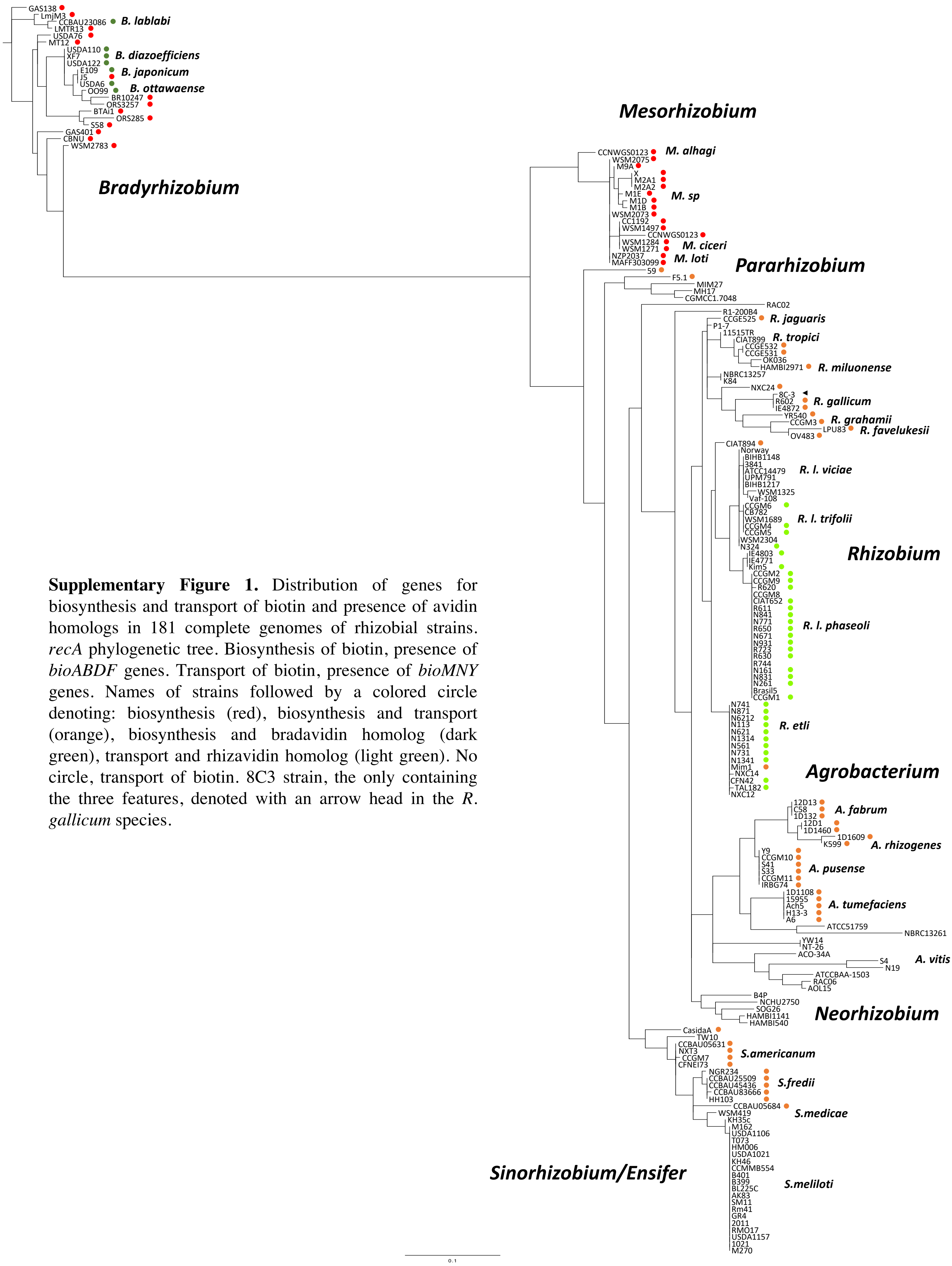

Supplementary Figure 1. Distribution of genes for biosynthesis and transport of biotin and presence of avidin homologs in 181 complete genomes of rhizobial strains. *recA* phylogenetic tree. Biosynthesis of biotin, presence of *bioABDF* genes. Transport of biotin, presence of *bioMNY* genes. Names of strains followed by a colored circle denoting: biosynthesis (red), biosynthesis and transport (orange), biosynthesis and bradavidin homolog (dark green), transport and rhizavidin homolog (light green). No circle, transport of biotin. 8C3 strain, the only containing the three features, denoted with an arrow head in the *R. gallicum* species.

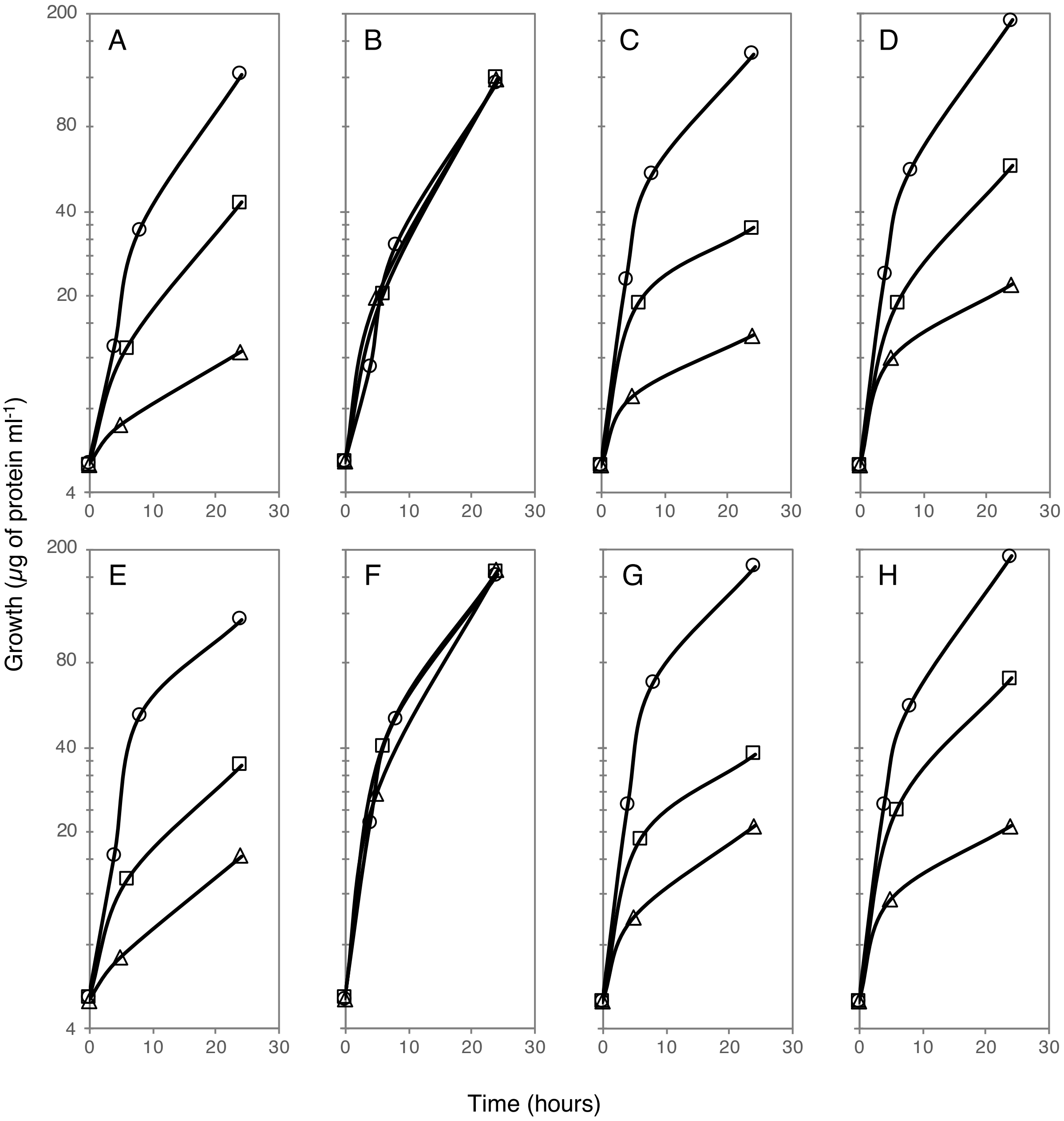

Supplementary Figure 2. Growth of rhizobial strains. Minimal medium of succinate-ammonium. Panels. A, *R. etli* CFN42 wild-type strain. B, CFN42 supplemented with 1 mM N-methyl aspartic acid. C, CFN42 supplemented with 1 mM aspartic acid. D, CFN42 supplemented with 10 mM aspartic acid. E, *R. phaseoli* CIAT652 wild-type strain. F, CIAT652 supplemented with 1 mM N-methyl aspartic acid. G, CIAT652 supplemented with 1 mM aspartic acid. H, CIAT652 supplemented with 10 mM aspartic acid. Symbols. First subculture, circles. Second subculture, squares. Third subculture, triangles. Average of three experiments. Deviations were less than 5%.

R. etli
CFN42 and tar-5 mutant

R. phaseoli
CIAT652 and 6-tar mutant

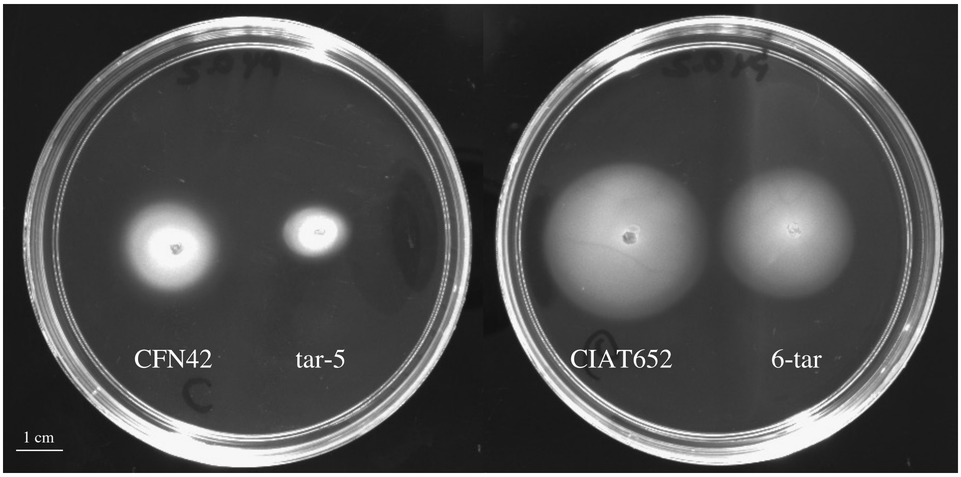

Medium	CFN42	tar-5	CIAT652	6-tar
	**Strains**			
PY	1.43 (0.51)	0.87 (0.15)	4.43 (0.86)	2.63 (0.15)
Succinate	1.50 (0.26)	1.10 (0.36)	1.83 (0.15)	1.43 (0.12)
Aspartate	1.67 (0.90)	1.67 (0.84)	3.03 (1.24)	2.27 (0.85)
Malate	1.67 (0.58)	1.33 (0.31)	2.07 (0.80)	1.63 (0.57)

Supplementary Figure 3. Determination of chemotaxis capability of wild-type and *tar* strains. Upper part. Example of cellular halos formed by strains. Lower part. Data of halo diameters, means (standard deviations) in cm. PY rich medium and minimal medium added with attractants at 0.1 mM (except for aspartic acid, at 1 mM) on soft agar 0.2% incubated by 72 h.
